# Anti-Cancer Efficacy of Silybin Derivatives - A Structure-Activity Relationship

**DOI:** 10.1371/journal.pone.0060074

**Published:** 2013-03-28

**Authors:** Chapla Agarwal, Ritambhara Wadhwa, Gagan Deep, David Biedermann, Radek Gažák, Vladimír Křen, Rajesh Agarwal

**Affiliations:** 1 Department of Pharmaceutical Sciences, Skaggs School of Pharmacy and Pharmaceutical Sciences, University of California San Diego, La Jolla, California, United States of America; 2 University of Colorado Cancer Center, Anschutz Medical Campus, University of Colorado, Aurora, Colorado, United States of America; 3 Institute of Microbiology, Academy of Sciences of the Czech Republic, Prague, Czech Republic; University of Washington, United States of America

## Abstract

Silybin or silibinin, a flavonolignan isolated from Milk thistle seeds, is one of the popular dietary supplements and has been extensively studied for its antioxidant, hepatoprotective and anti-cancer properties. We have envisioned that potency of silybin could be further enhanced through suitable modification/s in its chemical structure. Accordingly, here, we synthesized and characterized a series of silybin derivatives namely 2,3-dehydrosilybin (DHS), 7-*O*-methylsilybin (7OM), 7-O-galloylsilybin (7OG), 7,23-disulphatesilybin (DSS), 7-*O*-palmitoylsilybin (7OP), and 23-*O*-palmitoylsilybin (23OP); and compared their anti-cancer efficacy using human bladder cancer HTB9, colon cancer HCT116 and prostate carcinoma PC3 cells. In all the 3 cell lines, DHS, 7OM and 7OG demonstrated better growth inhibitory effects and compared to silybin, while other silybin derivatives showed lesser or no efficacy. Next, we prepared the optical isomers (A and B) of silybin, DHS, 7OM and 7OG, and compared their anti-cancer efficacy. Isomers of these three silybin derivatives also showed better efficacy compared with respective silybin isomers, but in each, there was no clear cut silybin A versus B isomer activity preference. Further studies in HTB cells found that DHS, 7OM and 7OG exert better apoptotic activity than silibinin. Clonogenic assays in HTB9 cells further confirmed that both the racemic mixtures as well as pure optical isomers of DHS, 7OM and 7OG were more effective than silybin. Overall, these results clearly suggest that the anti-cancer efficacy of silybin could be significantly enhanced through structural modifications, and identify strong anti-cancer efficacy of silybin derivatives, namely DHS, 7OM, and 7OG, signifying that their efficacy and toxicity should be evaluated in relevant pre-clinical cancer models in rodents.

## Introduction

**Figure 1:**
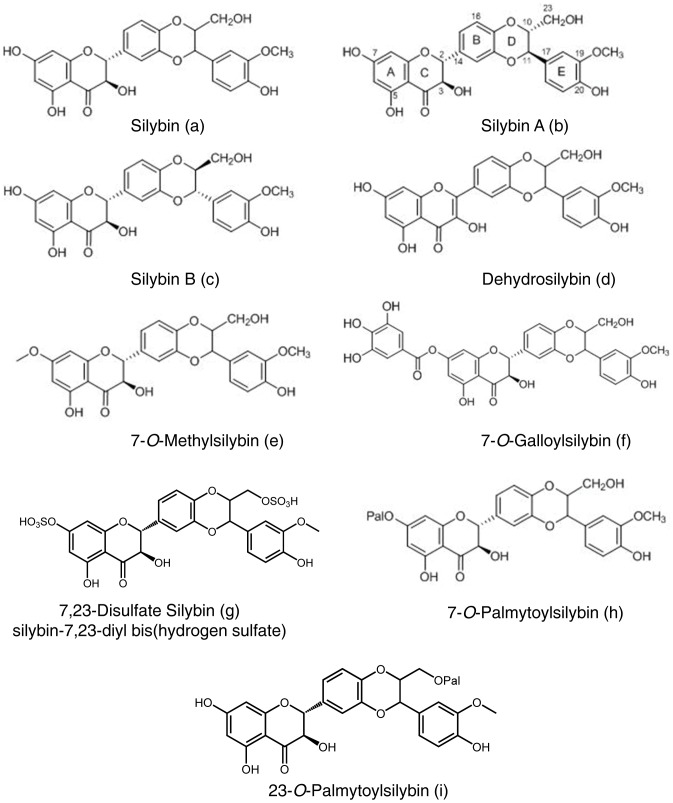
Chemical structure of silybin and its derivatives. (**a–h**) Chemical structures of silybin, silybin A, silybin B, 2,3-dehydrosilybin, 7-*O*-methylsilybin, 7-*O*-galloylsilybin, 7,23-disulphate silybin, 7-*O*-palmitoylsilybin, and 23-*O*-palmitoylsilybin.

Silybin or silibinin (C_25_H_22_O_10_, CAS No. 22888-70-6, Figure 1a) is a popular dietary supplement isolated from the seeds of *Silybum marianum* (L.) Gaertn (Family Asteraceae), known as milk thistle. Milk thistle extract denoted as silymarin has a long history of use in folk medicine and is now frequently employed for the prevention and/or treatment of liver disorders including viral hepatitis, liver cirrhosis associated with alcohol abuse, liver damage from drugs & industrial toxins [1]–[3]. Silybin is also considered an effective antidote against poisoning by death cap mushroom (*Amanita phalloides*) [2], [4]. Furthermore, silybin also exhibits strong antioxidant activity through scavenging hydroxyl radicals and inhibiting lipid peroxidation by acting as a chain-breaking antioxidant [5], [6]. In the past two decades, in addition to hepatoprotective and antioxidant effects, silybin has demonstrated remarkable anti-cancer as well as cancer chemopreventive efficacy in pre-clinical cell culture and animal models of several epithelial cancers including skin, bladder, colon, prostate, lung etc. [7], [8]. Based upon these promising results from pre-clinical studies, silybin has also been tested in human cancer patients in phase I-II pilot clinical trials, where it was reported to be well tolerated and showed plasma and target-tissue bioavailability [7], [9]–[11]. Several traditional toxicological tests have proven the non-toxic nature of silybin, and it is reported to be safe for human consumption [10], [12]. Based upon its proven as well as emerging preventive and therapeutic efficacy against cancer, it would be of high significance and translational value if the efficacy of silybin could be further enhanced through suitable chemical modification/s in its structure, as it is obvious that silybin has an effective “lead structure”.

In the recent years, there has been a greater emphasis on understanding the detailed chemical structure of silybin, identifying and synthesizing its stereoisomers as well as assessing their biological efficacy [13]–[17]. Silybin is now confirmed to be a 1∶1 mixture of two optical isomers namely silybin A and silybin B (Figure 1b and 1c). Furthermore, the detailed role of respective hydroxyl groups and moieties of silybin has been now well characterized, and that knowledge has permitted the identification of suitable sites for the designing and synthesis of novel silybin derivatives without affecting the biological activity of the resultant conjugates [18], [19]. These studies have identified that the most suitable positions for silybin modifications are 7-OH and/or 23-OH [18], [19]; and based upon these critical observations, several derivatives of silybin have been designed, synthesized and characterized [20]–[23]. For example, we have synthesized and characterized 7*-O*- and 23*-O*-acyl-derivatives of silibinin with varying acyl chain lengths, and have confirmed their antioxidant and anti-viral activities [20]. Similarly, a series of silybin galloyl esters have also been synthesized, and 7*-O*-galloylsilybin was identified as the most effective compound in terms of anti-angiogenic efficacy [21]. We have also reported that the anti-angiogenic efficacy of B isomer of 7*-O*-galloylsilybin is significantly higher than the A isomer [21]. Likewise, silybin oxidation product, 2,3-dehydrosilybin, has also been synthesized that showed better antioxidant potential than silybin; the carboxylic acid derivative of 2,3-dehydrosilybin, namely 2,3-dehydrosilybinic acid, has shown better anti-lipid-peroxidation and anti-radical scavenging activities and displays better water solubility [22], [24]. We have also prepared a large series of *O*-alkyl derivatives (methyl and benzyl) of silybin and 2,3-dehydrosilybin, and have identified some novel derivatives with strong efficacy against P-glycoprotein-mediated drug efflux activity [23]. Taken together, the above studies clearly suggested that the biological efficacy of silybin could be significantly enhanced through suitable chemical modifications.

With the view that several of silybin derivatives have shown better activity that parent molecule in various biological systems and that silybin itself exerts strong anti-cancer efficacy, in the present study, we compared the anti-cancer efficacy of several silybin derivatives (both existing as well as newly designed) in three different human cancer cell lines, and identified three silybin derivatives with anti-cancer activity better than silybin.

## Materials and Methods

### Cell Culture, Treatment and Reagents

Human bladder cancer HTB9 cells, colon cancer HCT116 cells and prostate carcinoma PC3 cells were purchased from the American Type Culture Collection (Manassas, VA, USA). HTB9 and PC3 cells were cultured in RPMI1640 medium supplemented with 10% fetal bovine serum and 100 U/mL penicillin G and 100 µg/mL streptomycin sulfate at 37°C in a humidified 5% CO_2_ incubator. HCT116 cells were grown in Dulbecco’s Modified Eagle Medium (DMEM) supplemented with 10% fetal bovine serum and 100 U/mL penicillin G and 100 µg/mL streptomycin sulfate at 37°C in a humidified 5% CO_2_ incubator. All the cell culture materials were from Invitrogen Corporation (Gaithersburg, MD). Cells were plated and treated at 40–50% confluency with different doses of silybin or its derivatives (5–60 µM in medium) dissolved originally in DMSO for the desired time periods under serum condition. An equal amount of DMSO (vehicle) was present in each treatment, including control; DMSO concentration did not exceed 0.1% (v/v) in any treatment. At the end of desired treatments, various analyses were performed as described later. Primary antibody for cPARP and anti-rabbit peroxidase conjugated secondary antibody were purchased from Cell Signaling (Beverly, MA). Antibody for tubulin was from Neomarkers (Fremont, CA). Hoechst 33342, crystal violet and propidium iodide (PI) were from Sigma-Aldrich (St. Louis, MO). ECL detection system and anti-mouse peroxidase conjugated secondary antibody were from GE Healthcare (Buckinghamshire, UK). All other reagents were obtained in their commercially available highest purity grade.

### Chemistry

Silymarin as a basic material for all consecutive products was obtained from Liaoning Senrong Pharm. Co., LTD (Panjin Liaoning Province, China). Optically pure silybin A and silybin B were obtained as described earlier [25], [26]; notably, a new preparative separation method developed by us recently [25], [26], which is based on lipase-catalyzed enzymatic discrimination of both silybin stereoisomers, enabled the preparation of all derivatives mentioned in this work in optically pure form at the large scale. The methods of synthesis, the synthetic schemes, and the analytical data confirming the structure of the previously synthesized and reported silybin derivatives (which were evaluated for their anti-cancer activity in the present study) are summarized in Methods S1 and Figures S1, S2, S3, S4, S5, and Tables S1, S2, S3, S4, S5, S6, S7, S8, respectively. Briefly, 2,3-Dehydrosilybin (DHS) A and B were prepared by a base-catalyzed oxidation of respective silybin A and B as described recently [22], [24]; 7-*O*-Methylsilybin (7OM) A and B were prepared by the base-catalyzed methylation of respective silybin A and B [23]; and 7-*O*-palmitoylsilybin (7OP), 23-*O*-palmitoylsilybin (23OP) and 7-*O*-galloylsilybin (7OG) from both silybin A and B were synthesized as described earlier by us [20]–[23].

### Preparation of 7,23-disulfate Silybin [silybin-7,23-diyl *bis* (Hydrogen Sulphate), DSS], and Characterization by Mass and NMR Spectra

To a stirred solution of silybin [silybin A, silybin B or natural silybin A&B (1∶1), 200 mg, 0.43 mmol] in DMF (2 mL), Py⋅SO_3_ (200 mg, 1.26 mmol) was added. The reaction was quenched after 1 h of stiring at 40°C by the addition of EtOH (0.5 mL). After a short stirring, the reaction mixture was loaded onto the column of silica gel and chromatographed with the solvent EtOH/MeOH/CH_2_Cl_2_/water (2/1/9/0.3) to yield a light yellow oil, which was lyophilized to obtain the title compound either as the pure diastereomer A or B or the mixture A&B (cca 160 mg, 60%) as a pale yellow lyophilizate.

The mass spectra of the synthesized compound were measured on a matrix-assisted laser desorption/ionization reflectron time-of-flight MALDI-TOF mass spectrometer ultraFLEX (Bruker-Daltonics, Bremen, DE). Positive spectra were calibrated externally using the monoisotopic [M+H]+ ion of human angiotensin I 1296.69* m/z*, MRFA peptide 524.26 *m/z* and CCA matrix peak 379.09 *m/z*. A 10 mg/mL solution of α-cyano-4-hydroxy-cinnamic acid or 2,5- dihydrobenzoic acid in 50% MeCN/0.3% acetic acid was used as a MALDI matrix. A 1 µL of sample dissolved in water was allowed to dry at ambient temperature and over-laid with a 0.3 µL of the matrix solution on the target. The MALDI-TOF positive or negative spectra were collected in reflectron mode. MS (MALDI-TOF): *m/z* (%) = 643 (17, M^+.^+H), 561 (32), 547 (36), 545 (100), 481 (25), 465 (69).

The NMR spectra were measured on Bruker AVANCE III 400 (observation frequence 400.13 MHZ for ^1^H, 100.62 MHz for ^13^C) in DMSO-*d_6_* (Sigma Aldrich) at 30°C. Residual solvent signal was used as internal standard ((δ_H_ 2.500, δ_C_ 39.60). Chemical shifts and interaction constants were recorded from spectra on which weighting functions (exponential with negative exponent and Gauss function) were applied. Chemical shifts are expressed in δ-scale (ppm) and interaction constants in Hz. Digital resolution allows for expressing chemical shifts of protons with three and carbons with two decimal places, respectively, and proton-proton interaction constants with one decimal place. Multiplicity of carbon signals was determined from proton-edited HSQC spectra. Signal assignment is based on 2D NMR experiments – COSY, HSQC, HMBC, which were performed using standard programs (Bruker BioSpin GmbH, Rheinstetten, DE). ^1^H and ^13^C NMR spectra of both optically pure silybinA-7,23-diyl *bis*(hydrogen sulphate) and silybinB-7,23-diyl *bis*(hydrogen sulphate), which have not been reported so far, are outlined in Tables 1 and 2, respectively.

**Table 1:** ^1^H NMR (400.13 MHz for ^1^H, 100.61 MHz for ^13^C, DMSO-*d_6_*, 30°C).

Atom #	Silybin A-7,23-diyl *bis*(hydrogen sulphate)	Silybin B-7,23-diyl *bis*(hydrogen sulphate)
**2**	5.077(d, 11.2)	5.076 (d, 11.4)
**3**	4.601 (dd, 6.1, 11.2)	4.593 (dd, 6.3, 11.4)
**6**	5.915 (d, 2.1)	5.912 (d, 2.1)
**8**	5.879 (d, 2.1)	5.882 (d, 2.1)
**10**	4.357 (ddd, 2.5, 5.8, 7.8)	4.350 (ddd, 2.6, 5.9, 7.8)
**11**	4.873 (d, 7.8)	4.876 (d, 7.8)
**13**	7.096 (d, 1.9)	7.088 (d, 1.9)
**15**	7.025 (dd, 1.9, 8.1)	7. 032 (dd, 1.9, 8.2)
**16**	6.991 (d, 8.1)	6.995 (d, 8.2)
**18**	7.034 (d, 1.9)	7.043 (d, 2.0)
**21**	6.802 (d, 8.1)	6. 799 (d, 8.1)
**22**	6.861 (dd, 1.9, 8.1)	6.864 (dd, 2.0, 8.1)
**23**	3.768 (dd, 2.5, 11.1)	3.765 (ddd, 2.6, 11.1)
	3.691 (dd, 5.8, 11.1)	3.692 (ddd, 5.9, 11.1)
**3-OH**	5.786 (d, 6.1)	5.783 (d, 6.3)
**5-OH**	11.881 (s)	11.886 (s)
**7-OH**	n.d.	n.d.
**19-OMe**	3.790 (s)	3.793 (s)
**20-OH**	9.141 (s)	9.120 (s)
**23-OH**	n.d.	n.d.

**Table 2:** ^13^C NMR (400.13 MHz for ^1^H, 100.61 MHz for ^13^C, DMSO-*d_6_*, 30°C).

Atom #	Silybin A-7,23-diyl *bis*(hydrogen sulphate)	Silybin B-7,23-diyl bis(hydrogen sulphate)
**2**	82.61	82.57
**3**	71.45	71.50
**4**	197.70	197.71
**4a**	100.46	100.48
**5**	163.35	163.34
**6**	96.18	96.15
**7**	167.08	166.99
**8**	95.16	95.13
**8a**	162.52	162.51
**10**	76.06	76.06
**11**	75.72	75.71
**12a**	143.18	143.14
**13**	116.59	116.70
**14**	130.27	130.28
**15**	121.51	121.31
**16**	116.36	116.40
**16a**	143.27	143.23
**17**	127.10	127.11
**18**	111.83	111.77
**19**	147.64	147.64
**20**	147.10	147.07
**21**	115.40	115.37
**22**	120.29	120.27
**23**	64.72	64.70
**19-OMe**	55.65	55.64

### Cell Growth Assays

In each case, cells were plated to about 40–50% confluency and treated with silybin or its derivatives or pure isomers of each compound under serum condition as detailed above. After 24 and 48 h of treatments, cells were collected by brief trypsinization and washed with PBS. Total cell number was determined by counting each sample in duplicate using a hemocytometer under an inverted microscope. Each treatment and time point had three independent plates.

### Apoptosis Assay

Quantitative apoptotic cell death by silybin and its derivatives in HTB9 cells was measured by Hoechst assay as described previously [27]. Briefly, after desired treatments, cells were collected and then stained with DNA-binding dye Hoechst 33342 and PI. Apoptotic cells were quantified using fluorescent microscope (Axioskope 2 plus-HBO 100, Zeiss, Jena, DE) by counting cells/microscopic field (at 400×) in six fields for each sample (in triplicate). Apoptotic cells showed bright blue (early apoptotic) or orange-red fluorescence (late apoptotic), which was distinguished from necrotic cells showing bright red fluorescence (PI-stained).

### Western Blotting

For Western blotting, lysates (80 µg) were denatured in 2X SDS-PAGE sample buffer and were resolved on 8% Tris-glycine gels. The separated proteins were transferred on to nitrocellulose membrane followed by blocking with 5% non-fat milk powder (w/v) in Tris-buffered saline (10 mM Tris–HCl, pH 7.5, 100 mM NaCl, 0.1% Tween 20) for 1 h at room temperature. After blocking, the membranes were probed with primary antibody for 2 h at room temperature and then overnight at 4°C followed by appropriate peroxidase-conjugated secondary antibody for 1 h at room temperature and visualized by ECL detection system. In each case, blots were subjected to multiple exposures on the film to make sure that the band density is in the linear range. For all results, autoradiogram/bands were scanned with Adobe Photoshop 6.0 (Adobe Systems Inc., San Jose, CA). To ensure equal protein loading, each membrane was stripped and re-probed with α-tubulin antibody.

### Clonogenic Assay

HTB9 cells were plated in 6-well plates and permitted to attach to the plates for 24 h. Thereafter, depending upon the treatment protocol, cells were treated with each compound every 72 h or treated only for 2 h every 24 h. At the end of indicated period, cells were washed twice with ice cold PBS, fixed with the mixture of methanol and glacial acetic acid (3∶1) for 10 minutes and then stained with 1% crystal violet in methanol for 15 minutes followed by washing with deionized water. Colonies with more than 50 cells were counted.

### Statistical Analysis

Statistical analysis was performed using SigmaStat 2.03 software (Jandel Scientific, San Rafael, CA). Data was analyzed using one way ANOVA followed by Tukey t-test, and a statistically significant difference was considered at *p*≤0.05.

## Results

### Effect of Silybin and its Derivatives on Cancer Cell Growth

First, we compared the growth inhibitory effect of silybin and its derivatives (chemical structures shown in Figure 1) DHS, 7OM, 7OG, DSS, 7OP and 23OP in human cancer cell lines from bladder, colon and prostate origin, namely HTB9, HCT116 and PC3 respectively; notably, silibinin has shown strong anti-cancer efficacy in pre-cancer models of bladder, colon and prostate cancers [7], [8]. As shown in Table 3, in HTB9 cells at 30 and 60 µM doses, DHS, 7OM and 7OG were more effective than silybin after 24 and 48 h of treatments in terms cell growth inhibition; however, at equimolar concentrations, the growth inhibitory effects of other derivatives, namely DSS, 7OP, and 23OP, were inconsistent and either equal or lower than silybin. Similarly in HCT116 cells, DHS, 7OM and 7OG were more effective in inhibiting cell growth compared to silybin and other silybin derivatives (Table 3). In PC3 cells, silybin derivative (30 and 60 µM doses) showed equal or better growth inhibition than silybin after 24 h of treatment (Table 3). Again, DHS, 7OM and 7OG were much more effective than silybin or other derivatives after 24 and 48 h of treatments in terms of cell growth inhibition; however the effect of other silybin derivatives (DSS, 7OP, and 23OP) was largely lost at 48 h time-point (Table 3). Together, these results clearly showed that only DHS, 7OM and 7OG derivatives of silybin have much stronger anti-cancer efficacy than parent structure, and therefore, only these three derivatives were used in subsequent studies.

**Table 3:** Effect of silybin and its derivatives on HTB9, HCT116, and PC3 cancer cell growth.

Treatments	Total cell no. x10^5^ (% growth inhibition)			
HTB9 cells	24 hrs		48 h	
	30 µM	60 µM	30 µM	60 µM
DMSO	2.5±0.1		4.7±0.1	
Silybin	2.0±0.1# (20%)	1.9±0.1* (24%)	3.9±0.3$ (17%)	3.9±0.3$ (17%)
2,3-dehydrosilybin	1.4±0.1* (44%)	1.1±0.0* (56%)	2.0±0.2* (57%)	1.2±0.1* (74%)
7-O-methylsilybin	1.6±0.1* (36%)	0.9±0.1* (64%)	1.3±0.4* (72%)	0.4±0.1* (91%)
7-O-galloylsilybin	1.4±0.2* (44%)	1.2±0.1* (52%)	1.9±0.3* (60%)	1.2±0.3* (74%)
7,23-disulphatesilybin	2.2±0.09 (12%)	2.3±0.1 (8%)	4.3±0.2 (8.5%)	3.8±0.3# (19%)
7-O-palmitoylsilybin	2.5±0.1 (0%)	2.5±0.2* (0%)	3.9±0.1$ (17%)	4.0±0.3 (15%)
23-O-palmitoylsilybin	1.8±0.1* (28%)	2.3±0.0 (8%)	4.6±0.3 (2%)	4.2±0.1 (11%)
**HCT116 cells**	**24 hrs**		**48 h**	
	**30 µM**	**60 µM**	**30 uM**	**60 µM**
DMSO	5.3±0.3		14.3±0.4	
Silybin	3.9±0.2 (26%)	4.3±0.2 (19%)	11.5±0.5$ (20%)	10.6±1.2* (26%)
2,3-dehydrosilybin	3.0±0.4* (43%)	2.2±0.3* (58%)	3.3±0.4* (77%)	2.1±0.3* (85%)
7-O-methylsilybin	3.0±0.1* (43%)	1.9±0.5* (64%)	6.4±0.6* (55%)	1.9±0.4* (87%)
7-O-galloylsilybin	3.3±0.1# (38%)	1.9±0.3* (64%)	4.2±0.4* (71%)	2.3±0.1* (84%)
7,23-disulphatesilybin	4.9±0.8 (8%)	4.8±0.3 (9%)	12.5±1.8 (13%)	14.5±0.1 (−1%)
7-O-palmitoylsilybin	5.1±0.4 (4%)	4.2±1.0 (21%)	14.1±0.6 (2%)	12.2±0.7 (15%)
23-O-palmitoylsilybin	3.4±0.6$ (36%)	4.6±0.7 (13%)	14.1±1.1 (2%)	11.2±1.4# (22%)
**PC3 cells**	**24 hrs**		**48 h**	
	**30 µM**	**60 µM**	**30 uM**	**60 µM**
DMSO	1.5±0.1		3.0±0.1	
Silybin	1.4±0.1 (7%)	1.2±0.2 (20%)	2.7±0.1 (10%)	1.6±0.1* (47%)
2,3-dehydrosilybin	0.7±0.2* (53%)	0.7±0.1* (53%)	0.7±0.1* (77%)	0.5±0.1* (83%)
7-O-methylsilybin	0.8±0.1* (47%)	0.8±0.1* (47%)	0.9±0.1* (70%)	0.7±0.2* (77%)
7-O-galloylsilybin	1.1±0.1* (27%)	0.8±0.0* (47%)	0.8±0.1* (73%)	0.8±0.2* (73%)
7,23-disulphatesilybin	1.1±0.0 (27%)	1.1±0.1* (27%)	3.0±0.2	2.5±0.0 (17%)
7-O-palmitoylsilybin	1.3±0.1 (13%)	1.1±0.3 (27%)	2.7±0.5 (10%)	2.0±0.1* (33%)
23-O-palmitoylsilybin	1.3±0.1 (13%)	1.3±0.1 (13%)	2.4±0.1# (20%)	2.2±0.0* (27%)

* p≤0.001;

# p≤0.01;

$ p≤0.05.

### Effect of Silybin and its Derivatives on Apoptosis Induction in HTB9 Cells

**Figure 2:**
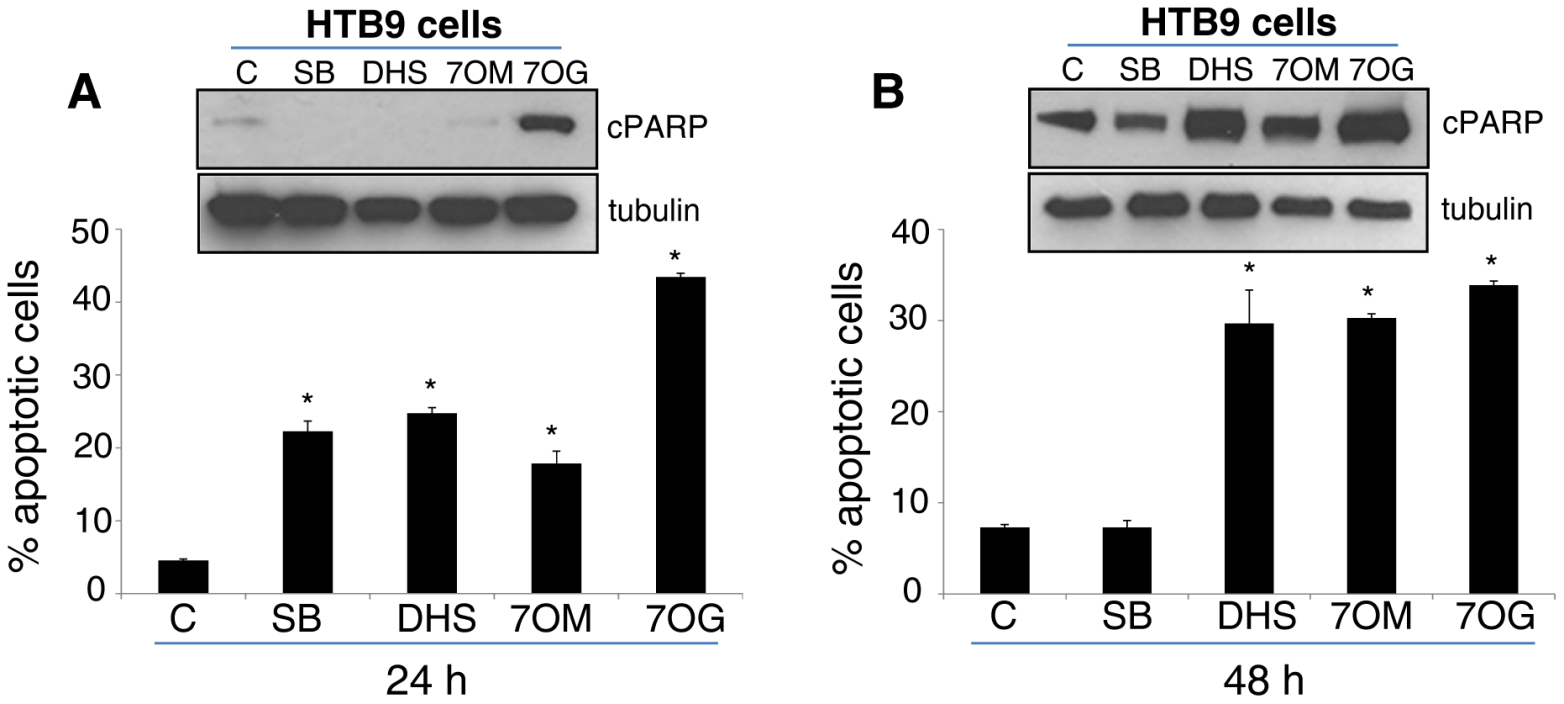
Effect of silybin and its derivatives on apoptosis in HTB9 cells. (**A–B**) HTB9 cells were treated with 30 µM dose of silybin (SB), 2,3-dehydrosilybin (DHS), 7-*O*-methylsilybin (7OM), and 7-*O*-galloylsilybin (7OG). After 24 or 48 h of each treatment, cells were collected and apoptotic population was determined by Hoechst assay as detailed in the ‘Materials and Methods’. In the bar diagrams, each data-point is representative of mean ± SD of 3 samples. (**A–B, western blot insert**) Cells were also collected following silybin or its derivatives treatment and analyzed for cPARP protein level by Western blotting. In each case, membranes were also stripped and re-probed with α-tubulin antibody to check protein loading. *p≤0.001.

Next, we compared the effect of silybin and its three most active derivatives (DHS, 7OM and 7OG) at 30 µM dose on the induction of apoptotic cell death in HTB9 cells. As shown in the figure 2A-bottom panel, after 24 h of treatment, silybin and its derivatives significantly increased the apoptotic cell death in HTB9 cell, and 7OG was most potent in inducing apoptosis. However, Western blot analyses for apoptosis marker cPARP showed a strong increase with only 7OG (Figure 2A-upper panel). The better efficacy of silybin derivatives was more clearly evident after 48 h of treatment where DHS, 7OM and 7OG significantly increased the apoptotic cell population (Figure 2B-bottom panel) as well as increased the level of cPARP in HTB9 cells (Figure 2B-upper panel). These results further confirmed the strong and better anti-cancer efficacy of DHS, 7OM and 7OG compared to silybin against HTB9 cells.

### Effect of Pure Optical Isomers of Silybin and its Derivatives on Cancer Cell Growth

As mentioned above that silybin is a mixture (1∶1 ratio) of optical isomers silybin A and silybin B, we next prepared large quantity of these two silybin isomers and utilized them to synthesize large quantities of their derivatives DHS (A and B), 7OM (A and B) and 7OG (A and B) as summarized in methods above. These pure optical isomers of silybin and their derivatives were then assessed for their cancer cell growth inhibitory effects. In all the three cancer cell lines, namely HTB9, HCT116 and PC3, pure isomers (30 and 60 µM doses) of DHS, 7OM and 7OG were more effective compared to respective isomer of silybin after 24 and 48 h of treatments (Table 4). When we compared the cell growth inhibitory effects of isomer A versus isomer B and their respective derivatives, no clear cut observation could be made, however, silybin B isomer and its derivatives seemed more effective than isomer A and its derivatives (Table 4).

**Table 4:** Effect of pure optical isomers of silybin, 2,3-dehydrosilybin, 7-*O*-methylsilybin, and 7-*O*-galloylsilybin on HTB9, HCT116, and PC3 cancer cell growth.

Treatments	Total cell no. x105 (% growth inhibition)			
HTB9 Cells	24 hrs		48 h	
	30 µM	60 µM	30 µM	60 µM
DMSO	2.5±0.1		5.5±0.2	
Silybin A	2.0±0.1# (20%)	2.0±0.3$ (20%)	4.7±0.8* (15%)	3.9±0.3* (29%)
2,3-dehydrosilybin A	1.8±0.1* (28%)	1.3±0.1* (48%)	1.8±0.1* (67%)	1.3±0.0* (76%)
7-O-methylsilybin A	1.5*±0.1* (40%)	1.2±0.1* (52%)	1.8±0.1* (67%)	0.5±0.1* (91%)
7-O-galloylsilybin A	1.7±0.1* (32%)	1.0±0.0* (60%)	1.5±0.1* (73%)	1.1±0.1* (80%)
Silybin B	2.2±0.1 (12%)	1.7±0.2* (32%)	4.2±0.3* (24%)	3.3±0.3* (40%)
2,3-dehydrosilybin B	1.3±0.1* (48%)	1.3±0.1* (48%)	1.5±0.1* (73%)	1.4±0.1* (75%)
7-O-methylsilybin B	2.1±0.1 (16%)	1.2±0.2* (52%)	2.2±0.1* (60%)	0.9±0.1* (84%)
7-O-galloylsilybin B	1.3±0.1* (48%)	0.8±0.2* (68%)	1.4±0.1* (75%)	0.9±0.1* (84%)
**HCT116 Cells**	**24 hrs**		**48 h**	
	**30 µM**	**60 µM**	**30 µM**	**60 µM**
DMSO	3.6±0.2		8.6±0.4	
Silybin A	3.4±0.3 (6%)	2.9±0.3# (19%)	8.7±0.4 (−1%)	7.0±0.3* (19%)
2,3-dehydrosilybin A	1.9±.0.1* (47%)	1.7±0.2* (53%)	1.7±0.1* (80%)	1.0±0.1* (88%)
7-O-methylsilybin A	2.0±0.3* (44%)	1.2±0.2* (67%)	1.1±0.1* (87%)	0.8±0.2* (90%)
7-O-galloylsilybin A	1.8±0.2* (50%)	1.0±0.1* (72%)	1.0±0.0* (88%)	0.6±0.1* (93%)
Silybin B	3.2±0.1 (11%)	3.3±0.1 (8.3%)	7.2±0.2* (16%)	6.1±0.6* (29%)
2,3-dehydrosilybin B	2.2±0.0* (39%)	1.2±0.1* (67%)	1.3±0.1* (85%)	1.2±0.1* (86%)
7-O-methylsilybin B	2.6±0.3* (28%)	1.3±0.1* (64%)	2.0±0.1* (77%)	1.0±0.1* (88%)
7-O-galloylsilybin B	1.5±0.1* (58%)	1.1±0.1* (69%)	0.8±0.2* (91%)	1.0±0.0* (88%)
**PC3 cells**	**24 hrs**		**48 h**	
	**30 µM**	**60 µM**	**30 µM**	**60 µM**
DMSO	2.2±0.10		4.5±0.2	
Silybin A	1.9±0.01 (14%)	1.9±0.1 (14%)	4.0±0.2 (11%)	3.7±0.3* (17.8%)
2,3-dehydrosilybin A	0.7±0.0* (68%)	0.1±0.1* (95%)	1.2±0.0* (73%)	1.3±0.1* (71%)
7-O-methylsilybin A	0.8±0.1* (64%)	1.1±0.1* (50%)	1.0±0.2* (78%)	0.9±0.0* (80%)
7-O-galloylsilybin A	1.7±0.4$ (23%)	0.7±0.1* (68%)	0.8±0.1* (82%)	1.1±0.1* (76%)
Silybin B	1.7±0.1$ (23%)	1.6±0.1* (27%)	3.6±0.2* (20%)	3.3±0.1* (27%)
2,3-dehydrosilybin B	1.1±0.1* (50%)	1.1±0.1* (50%)	1.1±0.1* (76%)	1.0±0.1* (78%)
7-O-methylsilybin B	1.2±0.2* (45%)	1.2±0.1* (45%)	1.2±0.3* (73%)	1.3±0.1* (71%)
7-O-galloylsilybin B	0.4±0.0* (82%)	0.3±0.1* (86%)	0.7±0.1* (84%)	0.4±0.1* (91%)

* p≤0.001;

# p≤0.01;

$ p≤0.05.

**Figure 3:**
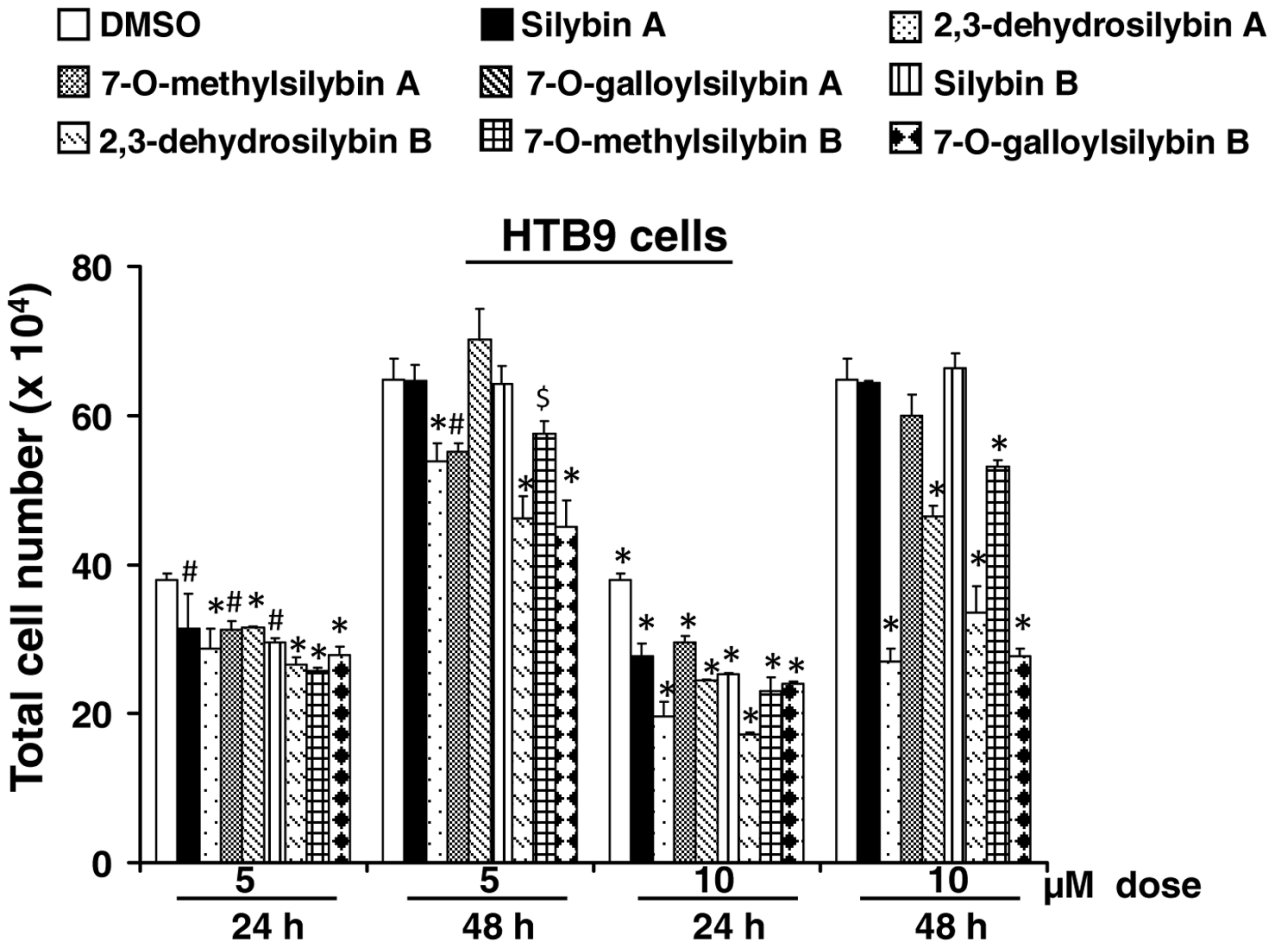
Effect of low doses of pure optical isomers of silybin and its derivatives on HTB9 cell growth. HTB9 cells were treated with 5 and 10 µM doses of silybin A, 2,3-dehydrosilybin A, 7-*O*-methylsilybin A, 7-*O*-galloylsilybin A or silybin B, 2,3-dehydrosilybin B, 7-*O*-methylsilybin B, 7-*O*-galloylsilybin B. After 24 or 48 h of each treatment, total cell number was determined as detailed in the ‘Materials and Methods’. In the bar diagrams, each data-point is representative of mean ± SD of 3 samples. *p≤0.001; #p≤0.01; $p≤0.05.

Next, we compared the growth inhibitory effects of lower doses (5 and 10 µM) of optical isomers of silybin (silybin A and silybin B) as well as its derivatives DHS (A and B), 7OM (A and B) and 7OG (A and B) in HTB9 cells. As shown in figure 3, at 5 µM dose, the difference between silybin and its derivatives was less striking. However, at 10 µM concentration, the difference in the efficacy of silybin isomers with their derivatives isomers was clearly apparent with stronger growth inhibition by derivatives compared to parent structures, specifically after 48 h of treatments (Figure 3). Together, these results suggested that similar to a better activity of racemic silybin isomers namely DHS, 7OM and 7OG compared to parent racemic silybin, the derivatives of pure silybin isomers A and B are also more effective compared to their respective pure silybin A and B isomers.

### Effect of Racemic Mixtures and Pure Optical Isomers of Silybin and its Derivatives on Colony Formation in HTB9 Cells

**Figure 4:**
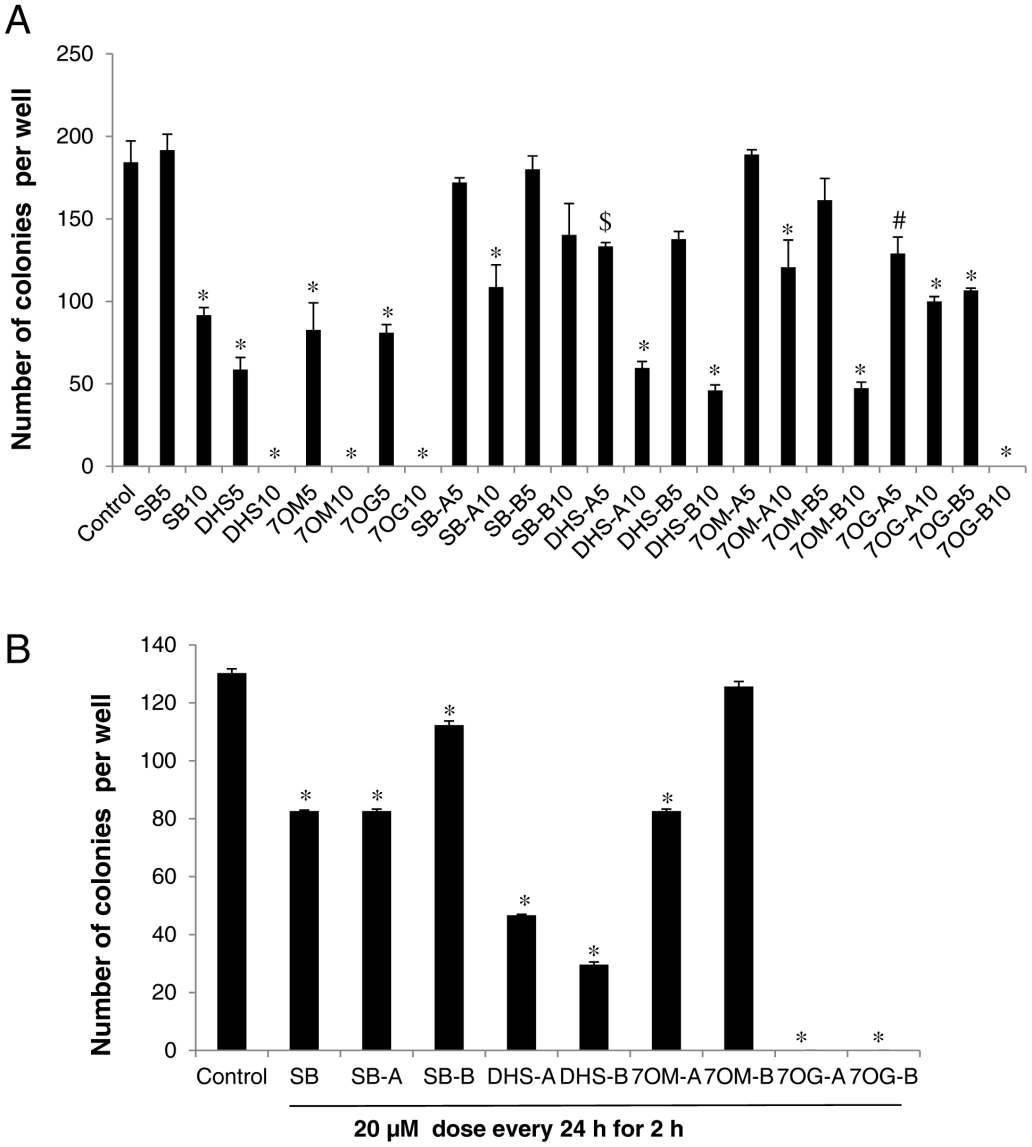
Effect of racemic mixtures and pure optical isomers of silybin and its derivatives on colony formation by HTB9 cells. (**A**) HTB9 cells (∼1000 cells) were plated in 6 well plates and treated every 72 h with 5 and 10 µM doses of silybin (SB), 2,3-dehydrosilybin (DHS), 7-*O*-methylsilybin (7OM), 7-*O*-galloylsilybin (7OG), silybin A (SB-A), 2,3-dehydrosilybin A (DHS-A), 7-*O*-methylsilybin A (7OM-A), 7-*O*-galloylsilybin A (7OG-A) or silybin B (SB-B), 2,3-dehydrosilybin B(DHS-B), 7-*O*-methylsilybin B (7OM-B), 7-*O*-galloylsilybin B (7OG-B). On 11^th^ day, cells were processed and colonies with more than 50 cells were counted. (**B**) HTB9 cells (∼1000 cells) were plated in 6 well plates and treated for 2 h every 24 h with 20 µM dose of silybin (SB), silybin A (SB-A), 2,3-dehydrosilybin A (DHS-A), 7-*O*-methylsilybin A (7OM-A), 7-*O*-galloylsilybin A (7OG-A) or silybin B (SB-B), 2,3-dehydrosilybin B(DHS-B), 7-*O*-methylsilybin B (7OM-B), 7-*O*-galloylsilybin B (7OG-B). After 7 days, cells were processed and colonies with more than 50 cells were counted. In the bar diagrams, each data-point is representative of mean ± SD of 3 samples. *, p≤0.001; #, p≤0.01; $, p≤0.05.

Next, we compared the effect of racemic mixtures and pure isomers of silybin and its three most effective DHS, 7OM and 7OG derivatives on colony formation by HTB9 cells. In the first experiment, we employed a dose of 20 µM of all the compounds and we observed a complete inhibition of colony formation by silybin as well as its derivatives (data not shown). Therefore, next we used lower doses of racemic mixtures as well as pure isomers of silybin and its derivatives. As shown in figure 4A, DHS, 7OM, and 7OG (5 and 10 µM doses every 72 h) inhibited the colony formation by HTB9 cells, and all three derivatives were more effective than silybin at both the doses. Similarly, treatment (5 and 10 µM doses) with pure isomers (A and B) of DHS, 7OM, and 7OG also strongly inhibited the colony formation by HTB9 cells; isomers of all the three derivatives were conclusively more effective compared to the respective isomers silybin A and silybin B (Figure 4A). Among all the pure isomers in this assay, 7OG-B showed the highest efficacy (Figure 4A).

In an another treatment regime, HTB9 cells were exposed to 20 µM dose of silybin or isomers of silybin and its derivatives (DHS, 7OM, and 7OG) for 2 h, and thereafter, fresh media was added without the compounds, and same treatment protocol was followed every 24 h. These experimental conditions were based upon our observation in completed clinical trial where we have reported silybin half-life and concentration in the plasma of cancer patients in the same range i.e. 20 µM silybin for about 2 h [28] As shown in figure 4B, under these treatment conditions, the efficacy of all the compounds was compromised to certain extent but still 7OG and DHS retained higher inhibitory efficacy against HTB9 colony formation. This assay confirmed that 7OG is the most effective among all the silybin derivatives followed by DHS and 7OM.

## Discussion

Cancer is one of the leading causes of mortality and morbidity around the world. In United States alone, a total of 1,638,910 new cancer cases and 577,190 deaths from cancer were projected to occur in 2012 [29]. With the increasing use of imaging tools and biomarkers, many cancers are diagnosed at early stages where therapeutic options are quite limited due to inherent side-effects associated with these treatments. In such patients, cancer chemoprevention and/or intervention through non-toxic agents such as silybin could be an attractive alternative option coupled with watchful-waiting. Furthermore, cancer chemotherapy is often limited by accompanying acute toxicity especially hepatotoxicity, which results in dose reductions or delays in therapy, potentially increasing the risk of a relapse [8]. Silybin has a long history of use for its hepatoprotective benefits, and therefore, it could be used along with chemotherapeutic agents as a hepatoprotectant [8]. Besides lowering toxicity, silybin could enhance the efficacy of cancer chemotherapeutic drugs [30]. In this regard, we have reported that silybin strongly synergizes human prostate carcinoma cells to doxorubicin-, cisplatin-, carboplatin-, and mitoxantrone-induced growth inhibition and apoptotic death [31]–[33]. Similar synergistic effects of silybin with doxorubicin and cisplatin have also been reported in various other cancer cell lines [34]–[37]. Other common elements contributing significantly towards carcinogenesis are chronic inflammation and oxidative stress [38]. Therefore, fruits and vegetables that are rich in antioxidants as well as anti-inflammatory agents are recommended for cancer chemoprevention strategies [38]. Silybin has proven anti-inflammatory and antioxidant properties [38], providing an additional rationale for its application in cancer prevention and intervention.

One of the major limitations, that have hindered silybin’s therapeutic utility in the past, is its bioavailability, which is largely due to its large multi-ring structure and low water solubility [22]. Several silybin formulations (phosphatidylcholine, nano-suspensions, etc.) have been tested to further improve its bioavailability and these formulations have shown significant improvement in silybin’s bioavailability [12]. For example, silybin’s formulation with phosphatidylcholine (known as ‘Silipide’, trade name ‘Siliphos’) was tested in prostate cancer and colon cancer patients and revealed high plasma bioavailability [9]–[11]. Another approach towards improving silybin’s bioavailability as well as biological efficacy is through suitable chemical modification/s in its structure; notably, silybin structure as well as the role of its functional moieties are well characterized in recent studies [13], [18], [19]. In that regard, we have already successfully synthesized and characterized a series of silybin derivatives, identified several of them with superior antioxidant and anti-angiogenic activities and found them water soluble, when compared with silybin [20]–[23]. Based on these important findings and on similar lines, here we synthesized and characterized another silybin derivative, namely 7,23-disulphate silybin (DSS), which is a potential silybin metabolite in Phase II biotransformation, and compared silibinin anti-cancer activity with DSS as well as several earlier synthesized silybin derivatives, such as DHS, 7OM, 7OG, 7OP, and 23OP. The major outcomes of these efficacy studies are: **a**) 7OG, DHS, 7OM consistently showed anti-cancer efficacy better than silybin, while silybin derivatives DSS, 7OP and 23OP showed lesser or inconsistent efficacy; supporting an overall structure-activity relationship; **b**) the pure optical isomers of 7OG, DHS, and 7OM also exerted better anti-cancer efficacy compared to respective silybin isomers (A and B); **c**) there was no clear cut preference between A and B isomers in terms of efficacy of silybin or its derivatives; and **d**) the overall efficacy of these silybin derivatives was dependent upon both the type of functional group present as well as their spatial localization.

In summary, the results from the present study showed that the anti-cancer efficacy of silybin could be significantly enhanced through suitable modifications in its chemical structure, and identified 7OG, DHS, and 7OM as more effective silybin derivatives. This finding is in line with previous observations showing that substitution at C-7 of silybin structure strongly improves its antioxidant and/or antiradical activity. Furthermore, since our results with three lead silybin derivatives were consistent in three entirely different human cancer cell lines, we suggest that the observed anti-cancer activity in not cell line specific. We would also like to emphasize here that these results are extremely important as silibinin has already been assessed in clinic for its safety and anti-cancer efficacy, and therefore, the three more effective silybin derivatives identified in the present study should be quickly studied in pre-clinical models as well as in clinic. In this direction, in future, we plan to assess the *in vivo* anti-cancer efficacy of these effective silybin derivatives as well as establish their toxicity (if any) and bioavailability. It will also be important to study that how the changes in the chemical structure of silybin affect its metabolism (glucuronidation, sulfation etc). Overall, the results from the present study are significant towards further enhancing the anti-cancer efficacy of silibinin as well as the effective use of silybin derivatives in cancer prevention and treatment.

## Supporting Information

Figure S1 **Preparative method for the production of optically pure silybin A (b) and silybin B (c).**
(TIF)

Figure S2 **Preparation of 2,3-Dehydrosilybin A (d).**
(TIF)

Figure S3 **Preparation of 7-** ***O*** **-Methylsilybin (e).**
(TIF)

Figure S4 **Preparation of 7-** ***O*** **-(3′,4′,5′-Tri-** ***O*** **-benzylgalloyl)silybin followed by that of 7-** ***O*** **-Galloylsilybin (f).**
(TIF)

Figure S5 **Preparation of 7-** ***O*** **-palmitoyl silybin (h) and 23-** ***O*** **-palmitoyl silybin (i).**
(TIF)

Table S1 **^13^C NMR data of DH-silybin A, B (600.23 MHz for ^1^H, 150.93 MHz for ^13^C, DMSO-** ***d_6_*** **, 30°C).**
(DOC)

Table S2 **^1^H NMR data of DH-silybin A, B (600.23 MHz for ^1^H, DMSO-** ***d_6_*** **, 30°C).**
(DOC)

Table S3 **^13^C NMR data (100.55 MHz, 30°C) of 7-O-Methylsilybin (e).**
(DOC)

Table S4 **^1^H NMR data (399.89 MHz, 30°C) of 7-** ***O*** **-Methylsilybin (e).**
(DOC)

Table S5 **^13^C NMR data of 7-** ***O*** **-Galloylsilybin (f) (DMSO-** ***d_6_*** **, 30°C).**
(DOC)

Table S6 **^1^H NMR data of 7-** ***O*** **-Galloylsilybin (f) (600.23 MHz for ^1^H, DMSO-** ***d_6_*** **, 30°C).**
(DOC)

Table S7 **^13^C NMR data of of 7-** ***O*** **-palmitoyl silybin (h) and 23-** ***O*** **-palmitoyl silybin (i) (100 MHz,** ***d_6_*** **-DMSO, 30°C).**
(DOC)

Table S8 **^1^H NMR data of of 7-** ***O*** **-palmitoyl silybin (h) and 23-** ***O*** **-palmitoyl silybin (i) (400 MHz,** ***d_6_*** **-DMSO, 30°C).**
(DOC)

Methods S1 **Methods for the preparation of the silybin derivatives.**
(DOCX)
