# Supplementary figures and images for: Anti-Cancer Efficacy of Silybin Derivatives - A Structure-Activity Relationship
Source: PLoS One. 2013 Mar 28;8(3):e60074. doi: 10.1371/journal.pone.0060074 (PMC3610875; doi:10.1371/journal.pone.0060074)

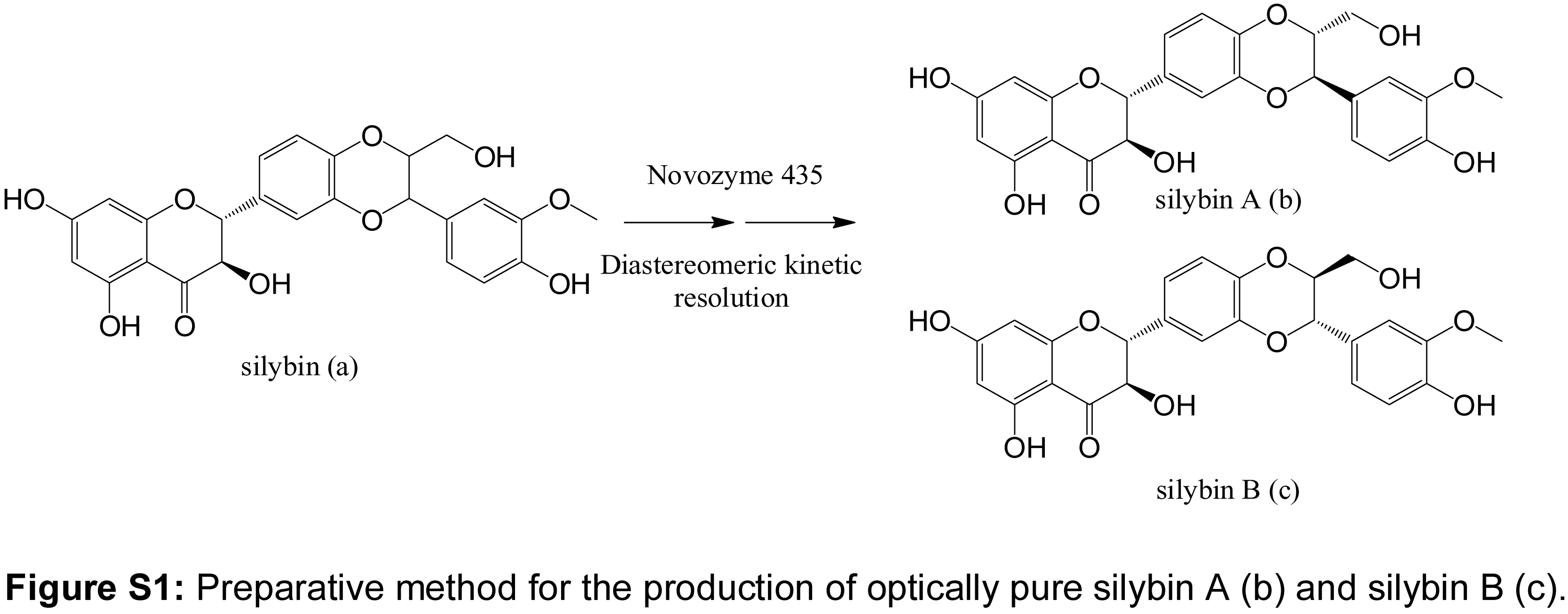

Supplement: Figure S1 — Preparative method for the production of optically pure silybin A (b) and silybin B (c). (TIF) [file pone.0060074.s001.tif]

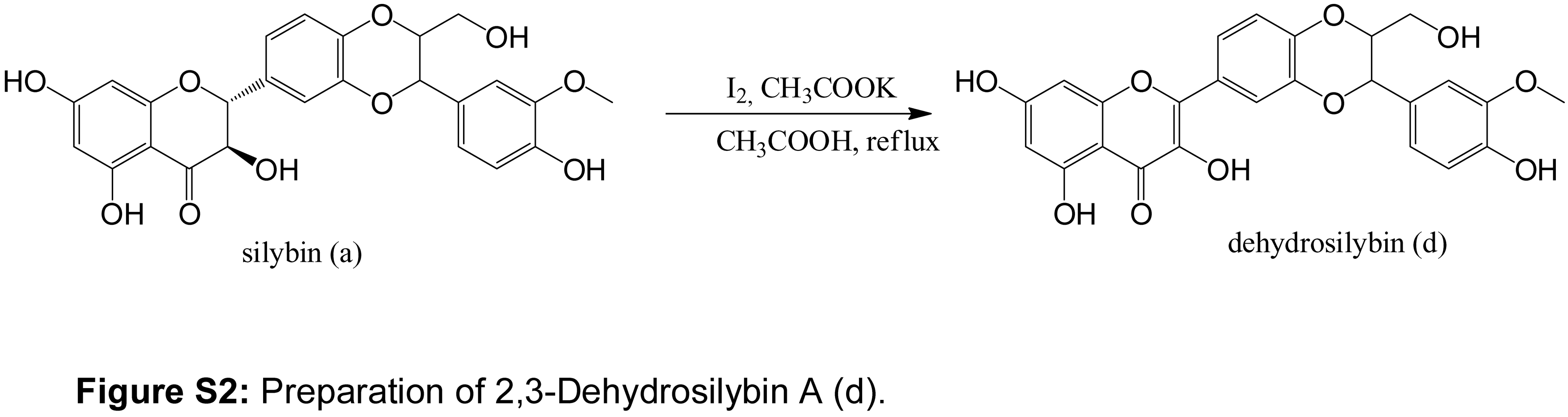

Supplement: Figure S2 — Preparation of 2,3-Dehydrosilybin A (d). (TIF) [file pone.0060074.s002.tif]

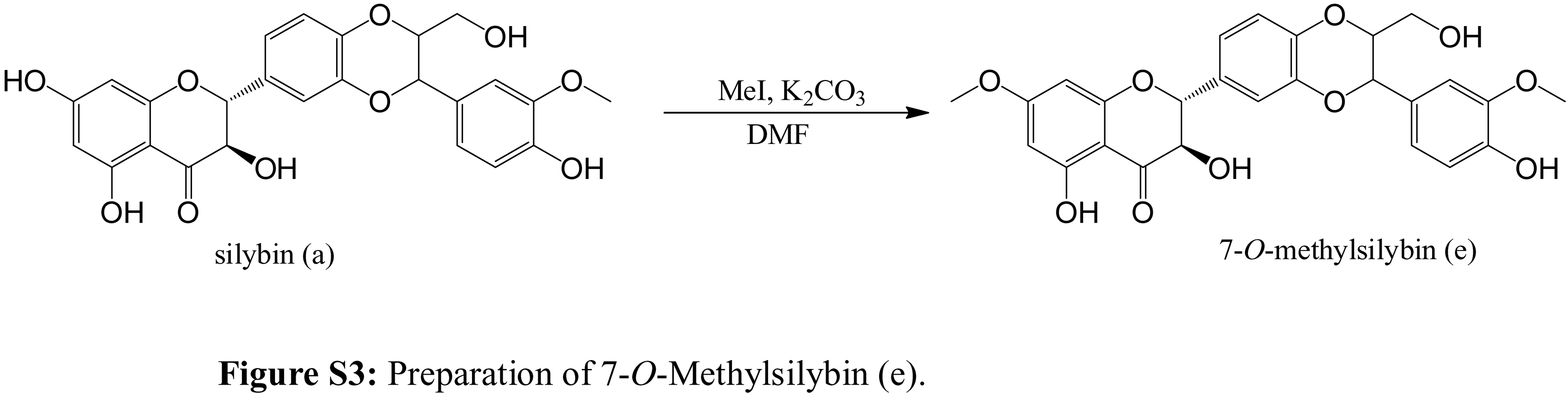

Supplement: Figure S3 — Preparation of 7- O -Methylsilybin (e). (TIF) [file pone.0060074.s003.tif]

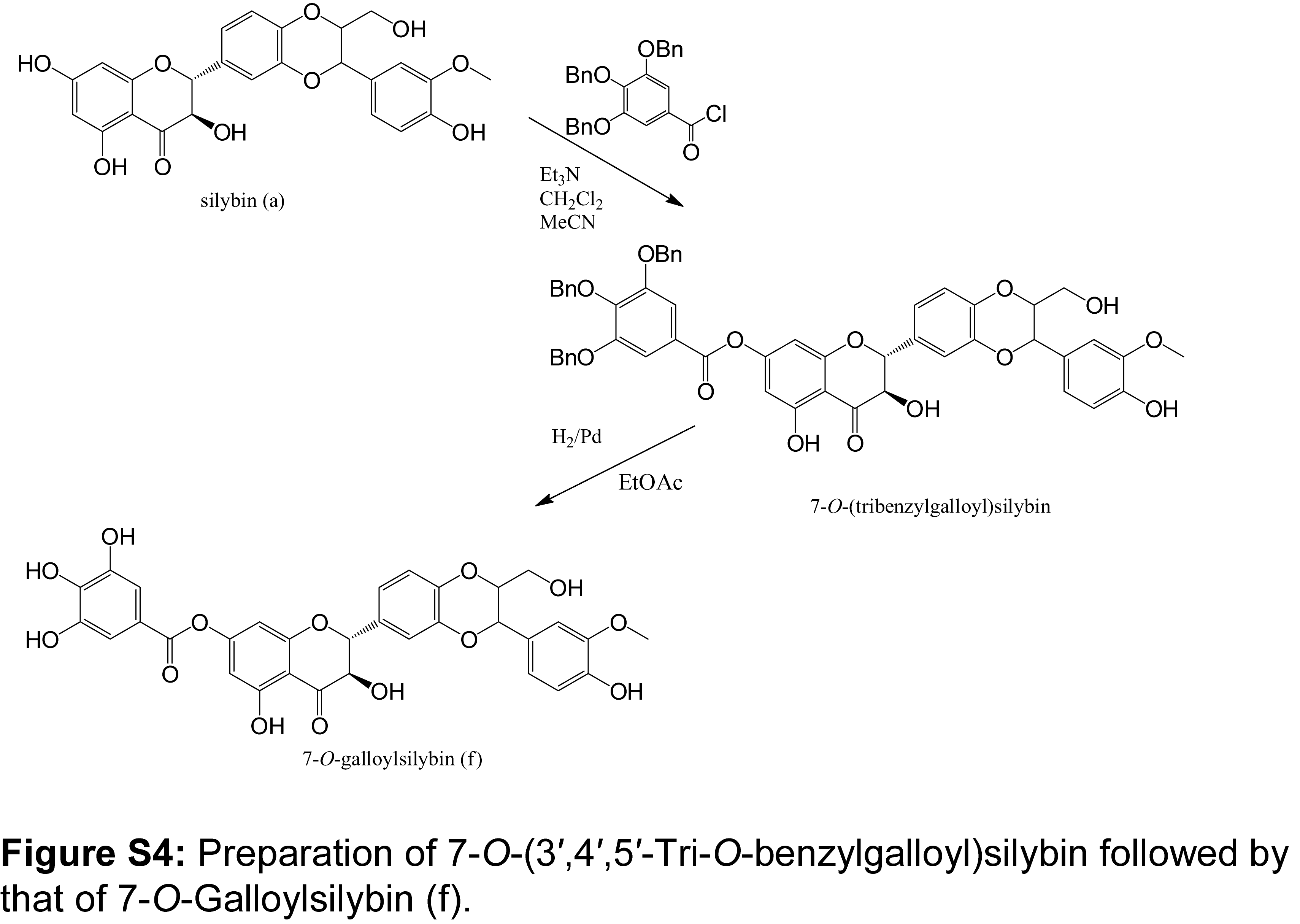

Supplement: Figure S4 — Preparation of 7- O -(3′,4′,5′-Tri- O -benzylgalloyl)silybin followed by that of 7- O -Galloylsilybin (f). (TIF) [file pone.0060074.s004.tif]

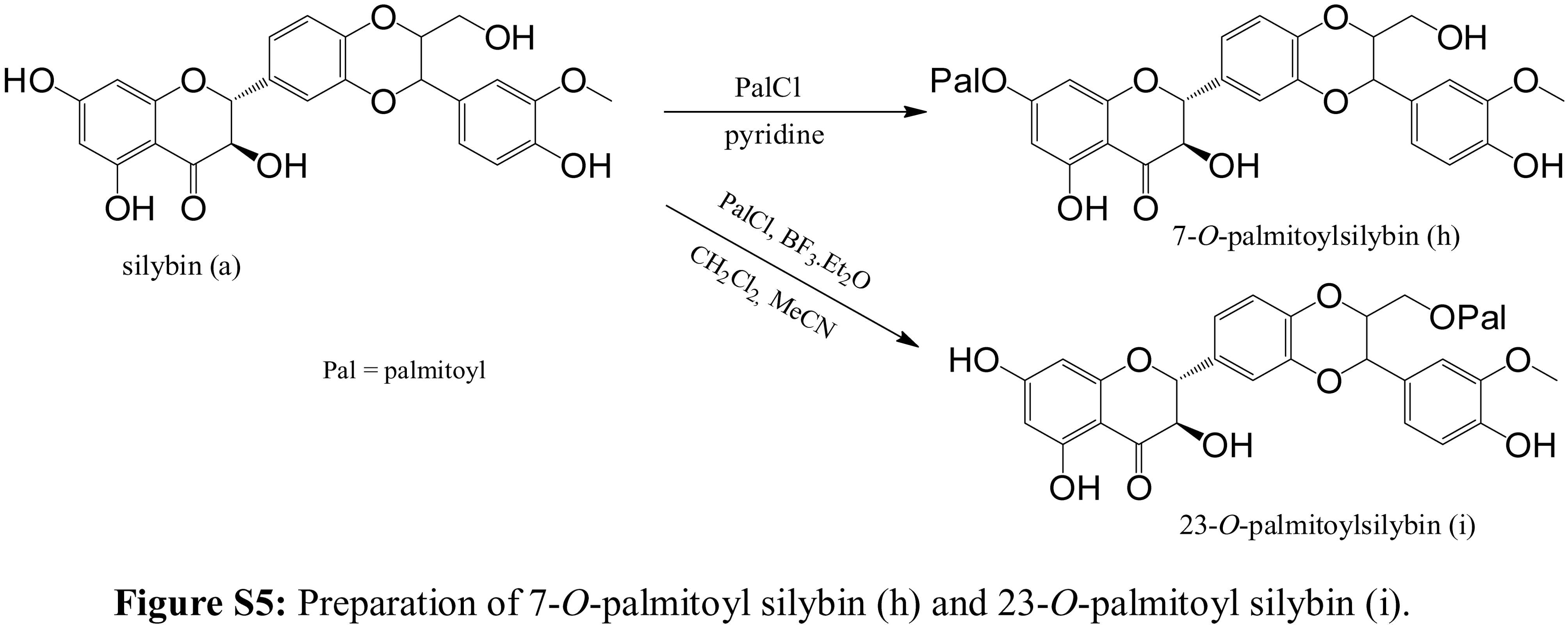

Supplement: Figure S5 — Preparation of 7- O -palmitoyl silybin (h) and 23- O -palmitoyl silybin (i). (TIF) [file pone.0060074.s005.tif]
