# Supplementary material for: Anti-Cancer Efficacy of Silybin Derivatives - A Structure-Activity Relationship
Source: PLoS One. 2013 Mar 28;8(3):e60074. doi: 10.1371/journal.pone.0060074 (PMC3610875; doi:10.1371/journal.pone.0060074)
Supplement: Table S1 — 13C NMR data of DH-silybin A, B (600.23 MHz for 1H, 150.93 MHz for 13C, DMSO- d6 , 30°C). (DOC) [file pone.0060074.s006.doc]

**Table S1: ^13^C NMR data of DH-silybin A, B (600.23 MHz for ^1^H, 150.93 MHz for ^13^C, DMSO-*d_6_*, 30 ^o^C).**

| Carbon | DH-silybin A | DH-silybin B |
| --- | --- | --- |
| 2 | 136.44 | 136.45 |
| 3 | 145.83 | 145.83 |
| 4 | 176.11 | 176.11 |
| 4a | 103.19 | 103.20 |
| 5 | 160.79 | 160.79 |
| 6 | 98.35 | 98.35 |
| 7 | 164.14 | 164.14 |
| 8 | 93.67 | 93.67 |
| 8a | 156.31 | 156.31 |
| 10 | 78.61 | 78.61 |
| 11 | 75.98 | 75.99 |
| 12a | 143.48 | 143.49 |
| 13 | 116.30 | 116.30 |
| 14 | 123.84 | 123.85 |
| 15 | 121.36 | 121.36 |
| 16 | 116.94 | 116.94 |
| 16a | 145.13 | 145.13 |
| 17 | 127.31 | 127.32 |
| 18 | 111.76 | 111.76 |
| 19 | 147.74 | 147.75 |
| 20 | 147.16 | 147.17 |
| 21 | 115.40 | 115.41 |
| 22 | 120.67 | 120.68 |
| 23 | 60.18 | 60.18 |
| 19-OMe | 55.78 | 55.78 |
