# Supplementary material for: Anti-Cancer Efficacy of Silybin Derivatives - A Structure-Activity Relationship
Source: PLoS One. 2013 Mar 28;8(3):e60074. doi: 10.1371/journal.pone.0060074 (PMC3610875; doi:10.1371/journal.pone.0060074)
Supplement: Table S2 — 1H NMR data of DH-silybin A, B (600.23 MHz for 1H, DMSO- d6 , 30°C). (DOC) [file pone.0060074.s007.doc]

**Table S2: ^1^H NMR data of DH-silybin A, B (600.23 MHz for ^1^H, DMSO-*d_6_*, 30^o^C).**

| Proton | DH-silybin A | DH-silybin B |
| --- | --- | --- |
| 2 | - | - |
| 3 | - | - |
| 6 | 6.191 (d, 2.1) | 6.192 (d, 2.1) |
| 8 | 6.459 (d, 2.1) | 6.458 (d, 2.1) |
| 10 | 4.274 (ddd, 2.5, 4.6, 8.0) | 4.273 (ddd, 2.6, 4.4, 8.0) |
| 11 | 4.962 (d, 8.0) | 4.961 (d, 8.0) |
| 13 | 7.767 (m) | 7.768 (d, 2.1) |
| 15 | 7.748 (m) | 7.754 (dd, 2.1, 8.5) |
| 16 | 7.119 (d, 8.4) | 7.118 (d, 8.5) |
| 18 | 7.044 (d, 2.0) | 7.045 (d, 1.7) |
| 21 | 6.814 (d, 8.0) | 6.815 (d, 8.0) |
| 22 | 6.888 (dd, 2.0, 8.0) | 6.888 (dd, 1.7, 8.0) |
| 23 | 3.567 (dd, 2.5, 12.4) | 3.568 (ddd, 2.6, 4.6, 12.0) |
|  | 3.363 (dd, 4.6, 12.4) | 3.363 (ddd, 4.4, 6.3, 12.0) |
| 3-OH | 9.579 (s) | 9.576 (s) |
| 5-OH | 12.416 (s) | 12.416 (s) |
| 7-OH | 10.819 (s) | 10.818 (s) |
| 19-OMe | 3.790 (s) | 3.791 (s) |
| 20-OH | 9.171 (s) | 9.171 (s) |
| 23-OH | 4.994 (br s) | 4.999 (dd, 4.6, 6.3) |
