# Supplementary material for: Anti-Cancer Efficacy of Silybin Derivatives - A Structure-Activity Relationship
Source: PLoS One. 2013 Mar 28;8(3):e60074. doi: 10.1371/journal.pone.0060074 (PMC3610875; doi:10.1371/journal.pone.0060074)
Supplement: Table S3 — 13C NMR data (100.55 MHz, 30°C) of 7-O-Methylsilybin (e). (DOC) [file pone.0060074.s008.doc]

**Table S3: ^13^C NMR data (100.55 MHz, 30 ^o^C) of 7-O-Methylsilybin (e).**

| Carbon | **7-*O*-Methylsilybin (e)** |
| --- | --- |
| 2 | 82.68 |
|  | 82.72 |
| 3 | 71.57 |
|  | 71.51 |
| 4 | 198.30 |
|  |  |
| 4a | 101.40 |
|  |  |
| 5 | 163.03 |
|  |  |
| 6 | 94.95 |
|  |  |
| 7 | 167.61 |
|  |  |
| 8 | 93.87 |
|  |  |
| 8a | 162.41 |
|  |  |
| 10 | 78.14 |
|  |  |
| 11 | 75.85 |
|  |  |
| 12a | 143.28 |
|  | 143.26 |
| 13 | 116.65 |
|  | 116.58 |
| 14 | 129.94 |
|  | 129.89 |
| 15 | 121.33 |
|  | 121.17 |
| 16 | 116.37 |
|  | 116.32 |
| 16a | 143.71 |
|  | 143.68 |
| 17 | 127.53 |
|  |  |
| 18 | 111.82 |
|  | 111.76 |
| 19 | 147.67 |
|  |  |
| 20 | 147.07 |
| 21 | 115.36 |
|  |  |
| 22 | 120.54 |
|  |  |
| 23 | 60.21 |
| 5-OMe | - |
| 7-OMe | 55.97 |
| 19-OMe | 55.75 |
|  | - |
| 20-OMe | - |
