# Supplementary material for: Anti-Cancer Efficacy of Silybin Derivatives - A Structure-Activity Relationship
Source: PLoS One. 2013 Mar 28;8(3):e60074. doi: 10.1371/journal.pone.0060074 (PMC3610875; doi:10.1371/journal.pone.0060074)
Supplement: Table S4 — 1H NMR data (399.89 MHz, 30°C) of 7- O -Methylsilybin (e). (DOC) [file pone.0060074.s009.doc]

**Table S4: ^1^H NMR data (399.89 MHz, 30 ^o^C) of 7-*O*-Methylsilybin (e).**

| Proton | **7-*O*-Methylsilybin (e)** |
| --- | --- |
| 2 | 5.136 d (11.4) |
| 3 | 4.668 dd (11.4,5.4) |
|  | 4.655 dd (11.4,5.4) |
| 6 | 6.188 d (2.2) |
|  |  |
| 8 | 6.100 d (2.2) |
|  | 6.094 d (2.2) |
| 10 | 4.176 ddd (7.9,4.7,2.5) |
|  | 4.166 ddd (7.9,4.7,2.5) |
| 11 | 4.915 d (7.9) |
|  |  |
| 13 | 7.099 d (1.8) |
|  | 7.092 d (1.8) |
| 15 | 7.028 dd (8.3,1.8) |
|  |  |
| 16 | 6.977 d (8.3) |
|  | 6.974 d (8.3) |
| 18 | 7.014 d (2.0) |
|  |  |
| 21 | 6.803 d (8.1) |
|  |  |
| 22 | 6.866 dd (8.1,2.0) |
|  |  |
| 23d | 3.546 ddd (12.2,3.4,2.5) |
|  |  |
| 23u | 3.351 ddd (12.2,4.7,3.4) |
|  |  |
| 5-OMe | - |
|  | - |
| 7-OMe | 3.788 s |
|  | 3.787 s |
| 19-OMe | 3.780 s |
| 20-OMe | - |
|  | - |
| 3-OH | 5.890 d (5.4) |
| 5-OH | 11.841 s |
|  |  |
| 7-OH | - |
| 20-OH | 9.122 s |
| 23-OH | 4.934 t (3.4) |
