# Supplementary material for: Anti-Cancer Efficacy of Silybin Derivatives - A Structure-Activity Relationship
Source: PLoS One. 2013 Mar 28;8(3):e60074. doi: 10.1371/journal.pone.0060074 (PMC3610875; doi:10.1371/journal.pone.0060074)
Supplement: Table S5 — 13C NMR data of 7- O -Galloylsilybin (f) (DMSO- d6 , 30°C). (DOC) [file pone.0060074.s010.doc]

**Table S5: ^13^C NMR data of 7-*O*-Galloylsilybin (f) (DMSO-*d_6_*, 30 ^o^C).**

| Carbon | **7-*O*-Galloylsilybin (f)** |
| --- | --- |
| 2 | 82.85  82.80 |
| 3 | 71.83  71.77 |
| 4 | 199.50  199.48 |
| 4a | 104.95 |
| 5 | 162.00 |
| 6 | 102.98 |
| 7 | 158.17 |
| 8 | 101.79 |
| 8a | 161.87  161.85 |
| 10 | 78.17  78.16 |
| 11 | 75.88 |
| 12a | 143.34  143.33 |
| 13 | 116.67  116.62 |
| 14 | 129.67  129.64 |
| 15 | 121.41  121.28 |
| 16 | 116.43  116.39 |
| 16a | 143.83  143.81 |
| 17 | 127.51  127.50 |
| 18 | 111.81  111.76 |
| 19 | 147.68  147.66 |
| 20 | 147.08  147.06 |
| 21 | 115.37  115.35 |
| 22 | 120.56  120.55 |
| 23 | 60.21 |
| 19-OMe | 55.75 |

Additional signals: 70.54 (*m*-OCH_2_), 74.34 (*p*-OCH_2_), 109.11 (C-*ortho*), 123.45 (C-*ipso*), 127.69 (C-*ortho'*), 128.23 (C-*ortho’’*), 127.99 (C-*para'*, C-*para''*), 128.13 (C-*meta''*), 128.23 (C-*ortho''*), 128.46 (C-*meta'*), 136.68 (C-*ipso'),* 137.20 (C-*ipso''*), 142.31 (C-*para*), 152.30 (C-*meta*), 163.10 (7-CO).
