# Supplementary material for: Anti-Cancer Efficacy of Silybin Derivatives - A Structure-Activity Relationship
Source: PLoS One. 2013 Mar 28;8(3):e60074. doi: 10.1371/journal.pone.0060074 (PMC3610875; doi:10.1371/journal.pone.0060074)
Supplement: Table S6 — 1H NMR data of 7- O -Galloylsilybin (f) (600.23 MHz for 1H, DMSO- d6 , 30°C). (DOC) [file pone.0060074.s011.doc]

**Table S6: ^1^H NMR data of 7-*O*-Galloylsilybin (f) (600.23 MHz for ^1^H, DMSO-*d_6_*, 30 ^o^C).**

| Proton | **7-*O*-Galloylsilybin (f)** |
| --- | --- |
| 2 | 5.271 (d, 11.5) |
| 3 | 4.804 (dd, 6.4, 11.5)  4.794 (dd, 6.4, 11.5) |
| 6 | 6.536 (d, 2.0) |
| 8 | 6.508 (d, 2.0)  6.502 (d, 2.0) |
| 10 | 4.182 (ddd, 2.8, 4.6, 7.8)  4.178 (ddd, 2.8, 4.6, 7.8) |
| 11 | 4.922 (d, 7.8) |
| 13 | 7.127 (d, 2.0)  7.122 (d, 2.1) |
| 15 | 7. 051 (dd, 2.1, 8.3)  7. 049 (dd, 2.0, 8.3) |
| 16 | 6.994 (d, 8.3)  6.993 (d, 8.3) |
| 18 | 7.022 (d, 2.2)  7.018 (d, 2.2) |
| 21 | 6. 808 (d, 8.0)  6. 807 (d, 8.0) |
| 22 | 6.872 (dd, 2.2, 8.0)  6.870 (ddd, 2.2, 6.0, 8.0) |
| 23 | 3.554 (ddd, 2.8, 6.0, 12.5)  3.546 (ddd, 2.8, 6.0, 12.5) |
|  | 3.362 (ddd, 4.6, 6.0, 12.5)  3.354 (dd, 4.6, 12.5) |
| 19-OMe | 3.781 (s) |
| 3-OH | 5.958 (d, 6.4)  5.956 (d, 6.4) |
| 5-OH | 11.711 (s) |
| 7-OH | - |
| 20-OH | 9.098 (s)  9.095 (s) |
| 23-OH | 4.915 (t, 6.0) |

Additional signals: 5.094 (2H, s, *p*-OCH_2_), 5.220 (4H, s, *m*-OCH_2_), 7.278 (2H, m, H-*meta''*), 7.290 (1H, m, H-*para''*), 7.349 (2H, m, H-*para'*), 7.370 (2H, m, H-*ortho'*'), 7.410 (4H, m, H-*meta'*), 7.501, 7.502 (2H, s, H-*ortho*), 7.528 (4H, m, H-*ortho*)*.*
