# Supplementary material for: Anti-Cancer Efficacy of Silybin Derivatives - A Structure-Activity Relationship
Source: PLoS One. 2013 Mar 28;8(3):e60074. doi: 10.1371/journal.pone.0060074 (PMC3610875; doi:10.1371/journal.pone.0060074)
Supplement: Table S7 — 13C NMR data of of 7- O -palmitoyl silybin (h) and 23- O -palmitoyl silybin (i) (100 MHz, d6 -DMSO, 30°C). (DOC) [file pone.0060074.s012.doc]

**Table S7: ^13^C NMR data of of 7-*O*-palmitoyl silybin (h) and 23-*O*-palmitoyl silybin (i) (100 MHz, *d_6_*-DMSO, 30 ^o^C).**

| C | **7-*O*-palmitoyl silybin (h)** | **23-*O*-palmitoyl silybin (i)** |
| --- | --- | --- |
| 2 | 82.77 | 82.52 |
|  | 82.57 | 82.47 |
| 3 | 71.81 | 71.54 |
|  | 71.52 | 71.47 |
| 4 | 199.47 | 197.65 |
|  |  | 197.61 |
| 4a | 104.80 | 100.45 |
| 5 | 163.34 | 163.36 |
| 6 | 102.92 | 96.15 |
| 7 | 158.10 | 167.01 |
| 8 | 101.66 | 95.11 |
| 8a | 161.98 | 162.48 |
|  | 161.84 |  |
| 10 | 78.19 | 75.06 |
|  |  |  |
| 11 | 75.90 | 75.96 |
| 12a | 143.50 | 143.25 |
|  | 143.32 | 143.22 |
| 13 | 116.64 | 116.80 |
|  |  | 116.67 |
| 14 | 129.68 | 130.57 |
|  |  | 130.54 |
| 15 | 121.31 | 121.54 |
|  | 121.30 | 121.32 |
| 16 | 116.38 | 116.44 |
|  |  | 116.37 |
| 16a | 143.80 | 143.21 |
|  | 143.67 | 143.09 |
| 17 | 127.54 | 126.63 |
|  |  |  |
| 18 | 111.80 | 111.78 |
|  |  | 111.73 |
| 19 | 147.69 | 147.83 |
|  |  | 147.82 |
| 20 | 147.16 | 147.40 |
|  | 147.07 |  |
| 21 | 115.38 | 115.45 |
|  |  |  |
| 22 | 120.56 | 120.65 |
|  |  |  |
| 23 | 60.23 | 62.42 |
| OMe | 55.74 | 55.74 |
| 1’ | 170.91 | 172.60 |
| 2’ | 33.52 | 33.27 |
| 3’ | 24.17 | 24.40 |
| 4’ | 28.30 | 28.44 |
| 5’ |  |  |
| 6’ |  |  |
| 7’ |  |  |
| 8’ |  |  |
| 9’ |  |  |
| 10’ |  |  |
| 11’ |  |  |
| 12’ |  |  |
| 13 |  |  |
| 14’ | 31.31 |  |
| 15’ | 22.11 |  |
| 16’ | 13.96 | 13.96 |

Additional signals – **h**: 29.04, 28.96, 28.84, 28.72, 28.63; **i**: 29.08, 29.07, 29.04, 29.01, 28.91, 28.73.
