# Supplementary material for: Anti-Cancer Efficacy of Silybin Derivatives - A Structure-Activity Relationship
Source: PLoS One. 2013 Mar 28;8(3):e60074. doi: 10.1371/journal.pone.0060074 (PMC3610875; doi:10.1371/journal.pone.0060074)
Supplement: Table S8 — 1H NMR data of of 7- O -palmitoyl silybin (h) and 23- O -palmitoyl silybin (i) (400 MHz, d6 -DMSO, 30°C). (DOC) [file pone.0060074.s013.doc]

**Table S8: ^1^H NMR data of of 7-*O*-palmitoyl silybin (h) and 23-*O*-palmitoyl silybin (i) (400 MHz, *d_6_*-DMSO, 30 ^o^C).**

| Proton | **7-*O*-palmitoyl silybin (h)** | **23-*O*-palmitoyl silybin (i)** |
| --- | --- | --- |
| 2 | 5.225 d (11.6) | 5.087 d (11.2) |
| 3 | 4.768 dd (11.6,6.4) | 4.595 dd (11.2,6.3) |
|  | 4.758 dd (11.6,6.4) | 4.582 dd (11.2,6.3) |
| 6 | 6.342 d (2.1) | 5.915 d (2.1) |
| 8 | 6.301 d (2.1) | 5.871 d (2.1) |
|  | 6.295 d (2.1) | 5.864 d (2.1) |
| 10 | 4.172 ddd (7.9,4.9,2.5) | 4.510 ddd (7.9,5.0,2.9) |
|  | 4.167 ddd (7.9,4.9,2.5) | 4.505 ddd (7.9,5.0,2.9) |
| 11 | 4.911 d (7.9) | 4.924 d (7.9) |
| 13 | 7.105 d (2.1) | 7.112 d (1.9) |
|  | 7.099 d (2.1) | 7.100 d (1.9) |
| 15 | 7.031 dd (8.3,2.1) | 7.031 dd (8.3,1.9) |
|  | 7.028 dd (8.3,2.1) | 7.022 dd (8.3,1.9) |
| 16 | 6.980 d (8.3) | 6.978 d (8.3) |
|  | 6.978 d (8.3) | 6.973 d (8.3) |
| 18 | 7.013 d (2.0) | 7.020 d (2.0) |
|  | 7.009 d (2.0) |  |
| 21 | 6.800 d (8.2) | 6,799 d (8,1) |
|  |  |  |
| 22 | 6.863 dd (8.2,2.0) | 6.857 dd (8.2,2.0) |
|  |  |  |
| 23d | 3.543 ddd (12.2,5.3,2.5) | 4.136 dd (12.3,2.9) |
|  |  | 4.132 dd (12.3,2.9) |
| 23u | 3.348 ddd (12.2,5.3,4.9) | 3.929 dd (12.3,5.0) |
|  |  |  |
| 19-OMe | 3.776 s | 3.774 s |
|  |  | 3.771 s |
| 3-OH | 5.937 d (6.4) | 5.794 d (6.3) |
| 5-OH | 11.890 s | 11.870 s |
| 7-OH | - | 10.810 br s |
| 20-OH | 9.128 s | 9.182 s |
| 23-OH | 4.953 t (5.3) | - |

Additional signals **h**: 2.544 (t, 2 H, *J* = 7.3 Hz, 2 × H-2‘), 1.600 (m, 2 H, 2 × H-3‘), 0.847 (t, 3 H, *J* = 7.0 Hz, 3 × H-16‘). **i**: 2.298 (m, 2 H, 2 × H-2‘), 1.945 (m, 2 H, 2 × H-3‘), 1.240 (m, 2 H, 2 × H-4‘), 0.843 (t, 3 H, *J* = 7.0 Hz, 3 × H-16‘).
