# Supplementary material for: Anti-Cancer Efficacy of Silybin Derivatives - A Structure-Activity Relationship
Source: PLoS One. 2013 Mar 28;8(3):e60074. doi: 10.1371/journal.pone.0060074 (PMC3610875; doi:10.1371/journal.pone.0060074)
Supplement: Methods S1 — Methods for the preparation of the silybin derivatives. (DOCX) [file pone.0060074.s014.docx]

**Methods S1**

**Preparative method for production of optically pure silybin A (b)**^1^

Schematic of preparation is shown in Figure S1. Novozym 435 (4.5 g, ≥10,000 U/g, 15% (w/w) (catalyst/substrate)) was added to a solution of 23-*O*-acetylsilybin (30 g; 57.2 mmol) in MTBE/n-BuOH, 9:1 (1.2 L) and the mixture was shaken at 45 °C and 170 rpm for 42 h. After enzyme removal, the solution was evaporated and the crude mixture was purified by column chromatography (CHCl_3_/acetone/HCOOH, 90:10:1), yielding silybin B (**c**, 17.93 g; 65%, d.e. = 44%), which was used in the subsequent procedure for further enzymatic enrichment, and 23-*O*-acetylsilybin A (9.73 g; 32%, d.e. = 95%).

The resulting 23-*O*-acetylsilybin A (9.73 g; 18.55 mmol) was dissolved in MTBE/n-BuOH, 9:1 (600 mL), Novozym 435 (9.73 g, ≥10,000 U/g, 100% (w/w) (catalyst/substrate)) was added and the mixture shaken at 35 °C and 170 rpm for 100 h. The enzyme was filtered off, solvents were evaporated and the crude mixture was crystallized from MeOH/H_2_O to yield the first portion of highly pure silybin A (**b**, 3.7 g; 41%, d.e. = 98%).

The second crystallization of the mother liquor yielded crystalline silybin A (**b**) with a somewhat lower purity (1.71 g; 19%, d.e. = 84%).

The resulting mother liquor was evaporated and purified by column chromatography (CHCl_3_/acetone/HCOOH, 90:10:1) yielding the final portion of silybin A (**b**) (2.58 g; 29%, d.e. = 95%).

The total yield of silybin A (**b**) with d.e. ≥95% was 6.28 g (45.6% from silybin). NMR and MS data were in accordance with those published previously.^2^

**Preparative method for production of optically pure silybin B (c)**^1^

Schematic of preparation is shown in Figure S1. Optically enriched silybin B (**c**, 17.93 g; 37.17 mmol, d.e. = 44%) obtained from the large-scale preparation of silybin A (**b**) was enzymatically acetylated as previously described, yielding 23-O-acetylsilybin B (19.5 g; 37.17 mmol; 100%, d.e. = 44%), which was again treated with Novozym 435 (5.85 g; 30% (w/w) (catalyst/substrate)) in toluene/n-BuOH, 9:1 (900 mL) and the mixture shaken at 45 °C and 170 rpm for 48 h.

The enzyme was removed by filtration and the filtrate evaporated to yield silybin B (**c**, 10.04 g; 56%, d.e. = 95%) after chromatography (CHCl_3_/acetone/HCOOH, 90:10:1), as well as 23-O-acetylsilybin A (7.02 g; 36%, d.e. = 36%), which can be recuperated for further enrichment, e.g., using the above method for silybin A preparation.

**Preparation of 2,3-Dehydrosilybin A (d)**^3^

Schematic of preparation is shown in Figure S2. Silybin (**a**; 2 g, 4.146 mmol), iodine (2 g, 15.760 mmol), and potassium acetate (12 g, 122.26 mmol) were dissolved in glacial acetic acid (100 mL) and stirred under reflux for 4 h. The mixture was then poured into ice-cold water (500 mL) and the precipitate formed was filtered off, washed thoroughly with water till neutral pH and then dissolved in ethyl acetate. EtOAc solution was dried over anhydrous Na_2_SO_4_ and evaporated to dryness. The evaporated residue was dissolved in EtOH (84 mL) and conc. HCl (16 mL) was added. Resulting solution was stirred under reflux for 1 h, then it was poured into ice-cold water (500 mL), precipitate formed was filtered off, washed till neutral pH, and the crude product on the filter was dissolved in acetone. The solution obtained was evaporated to dryness by co-evaporation with absolute EtOH. The crude product was re-crystallized from MeOH/H_2_O (9:1, v/v; 3 times) to give pure title compound **d** (880 mg, 44%). [α] = -53.1 (acetone, c = 0.356 g/100 mL). MS (ACPI+/- measured on MICROMAS LC-MS PLATFORM in MeOH) for C_25_H_20_O_10_ calcd. 480.4322 [M^+.^ 480]: 479 (M^+.^-H). The ^13^C NMR data of DH-silybin A, B are shown in Table S1 and the ^1^H NMR data of DH-silybin A, B are shown in Table S2.

**Preparation of 7-*O*-Methylsilybin (e)**^4^

Schematic of preparation is shown in Figure S3. K_2_CO_3_ (210 mg; 1.520 mmol) and MeI (0.1 mL, 1.606 mmol) were added to a stirred mixture of silybin (**a**) (530 mg, 1.099 mmol) in dry acetone (45 mL) and the reaction mixture was refluxed for 3.5 h under argon. The reaction was quenched by the addition of concd HCl (1 mL) and after short stirring the resulting mixture was diluted with water (150 mL) and extracted with ethyl acetate (2× 50 mL). The organic layer was dried (Na_2_SO_4_) and evaporated. The residue afforded after flash chromatography (chloroform/acetone/toluene/formic acid, 85:10:5:1) 7-*O*-methylsilybin (**e**) (290 mg, 53%) as a white amorphous solid. MS-ESI (*m*/*z*): 497 [M + H]^+^. The ^13^C NMR data of 7-*O*-methylsilybin (**e**) are shown in Table S3 and the ^1^H NMR data of 7-*O*-methylsilybin (**e**) are shown in Table S4.

**Preparation of 7-*O*-(3′,4′,5′-Tri-*O*-benzylgalloyl)silybin followed by that of 7-*O*-Galloylsilybin (f)**^5^

Schematic of preparation is shown in Figure S4. Et_3_N (0.145 mL, 1.042 mmol) was added to a stirred solution of silybin (**a**, 250 mg, 0.518 mmol) in CH_2_Cl_2_/MeCN (20 mL, 1:3 v/v), and the mixture was stirred at room temperature for 10 min. Then a solution of tribenzylgalloylchloride (287 mg, 0.626 mmol) in CH_2_Cl_2_ (10 mL) was added dropwise and the resulting mixture was stirred at room temperature for 1 h. Solvents were evaporated to dryness by co-evaporation with toluene. The dry solid left after flash chromatography (CHCl_3_/acetone/HCO_2_H, 95:5:1) was the title compound  (380 mg, 81%) as a white amorphous solid. MS-ESI (*m*/*z*): 927 (M^+^ + Na)

7-*O*-(3′,4′,5′-Tri-*O*-benzylgalloyl)silybin (500 mg, 0.553 mmol) was dissolved in EtOAc (30 mL), and then Pd/C (500 mg, 10% Pd) was added. The reaction mixture was stirred under a hydrogen atmosphere for 12 h. The Pd was then removed by filtration through Celite that was washed with acetone, and the solvent was evaporated. The residue left after flash chromatography (CHCl_3_/acetone/HCO_2_H, 5:1:0.1) was the title compound (**f**) (320 mg, 91%) as a white amorphous solid. HRMS calcd (M – H^+^) 633.1244, found 633.1243. The ^13^C NMR data of 7-*O*-Galloylsilybin (**f**) are shown in Table S5 and the ^1^H NMR data of 7-*O*-Galloylsilybin (**f**) are shown in Table S6.

**Preparation of 7-*O*-palmitoyl silybin (h) and 23-*O*-palmitoyl silybin (i)**^6^

Schematic of preparation is shown in Figure S5. Palmitoyl chloride (0.311 mmol, 1.5 equiv) was added to a cooled solution (0 ° C) of silybin (**1**; 100 mg, 0.207 mmol) in dry pyridine (7 mL) and the reaction mixture stirred at 0° C for 1 h. The reaction was stopped by diluting the reaction mixture with ice-cold HCl (50 mL, 5% solution in water, v/v) and after brief stirring the solution extracted with ethyl acetate (3 × 30 mL), the organic phase washed with brine, dried over anhydrous Na_2_SO_4_ and evaporated under reduced pressure. Flash chromatography on silica gel (chloroform/acetone/formic acid 95:5:1) yielded the 7-*O*-palmitoyl silybin (**h**).

Similarly, for the preparation of 23-*O*-palmitoyl silybin (i), Silybin (**a**;100 mg; 0.207 mmol) was dissolved in a CH_3_CN/CH_2_Cl_2_ mixture (1:1, 10 mL, v/v). Palmitoyl chloride (0.207 mmol, 1 equiv) and BF_3_∙Et_2_O (0.062 mL, 0.248 mmol, 50% solution in diethyl ether; v/v) were added and the mixture was stirred for 2 h at 0° C; then additional BF_3_·Et_2_O (0.062 mL, 0.248 mmol, 50% solution in diethyl ether, v/v) was added and the stirring continued for 1 h at room temperature. The mixture was then diluted with a saturated ice-cold solution of NaHCO_3_ (15 mL) and briefly stirred (approx. 10 min). The solution was extracted with ethyl acetate (2 × 15 mL), the organic phase washed with brine, dried over anhydrous Na_2_SO_4_ and evaporated under reduced pressure. Flash chromatography on silica gel (chloroform/acetone/formic acid 95:5:1) yielded the corresponding 23-*O*-palmitoyl silybin (**i**). The ^13^C NMR data of 7-*O*-palmitoyl silybin (**h**) and 23-*O*-palmitoyl silybin (**i**) are shown in Table S7 and the ^1^H NMR data of 7-*O*-palmitoyl silybin (**h**) and 23-*O*-palmitoyl silybin (**i**) are shown in Table S8.

**References**

1. Gažák, R.; Marhol, P.; Purchartova, K.; Monti, D.; Biedermann, D.; Riva, S.; Cvak, L.; Křen, V. Large-scale separation of silybin diastereoisomers using lipases. *Process Biochem.* **2010,** 45, 1657-1663.

2. Křen, V.; Gažák, R.; Purchartová, K.; Marhol, P.; Biedermann, D.; Sedmera, P. Chemoenzymatic preparative separation of silybins A and B. *J. Mol. Catal. B-Enzym.* **2009,** 61, 247-251.

3. Gažák, R.; Trouillas, P.; Biedermann, D.; Fuksová, K.; Marhol, P.; Kuzma, M.; Křen, V. Base-catalyzed oxidation of silybin and isosilybin into 2,3-dehydro derivatives. *Tetrahedron Lett.* 54, 315-317.

4. Džubák, P.; Hajduch, M.; Gažák, R.; Svobodová, A.; Psotová, J.; Walterová, D.; Sedmera, P.; Křen, V. New derivatives of silybin and 2,3-dehydrosilybin and their cytotoxic and P-glycoprotein modulatory activity. *Biorg. Med. Chem.* **2006,** 14, 3793-3810.

5. Gažák, R.; Valentová, K.; Fuksova, K.; Marhol, P.; Kuzma, M.; Medina, M. A.; Oborna, I.; Ulrichova, J.; Kren, V. Synthesis and Antiangiogenic Activity of New Silybin Galloyl Esters. *J. Med. Chem.* 54, 7397-7407.

6. Gažák, R.; Purchartová, K.; Marhol, P.; Živná, L.; Sedmera, P.; Valentová, K.; Kato, N.; Matsumura, H.; Kaihatsu, K.; Křen, V. Antioxidant and antiviral activities of silybin fatty acid conjugates. *Eur. J. Med. Chem.* 45, 1059-1067.
